# IL-1 Receptor Blockade Alleviates Graft-versus-Host Disease through Downregulation of an Interleukin-1**β**-Dependent Glycolytic Pathway in Th17 Cells

**DOI:** 10.1155/2015/631384

**Published:** 2015-12-20

**Authors:** Min-Jung Park, Seung Hoon Lee, Sung-Hee Lee, Eun-Jung Lee, Eun-Kyung Kim, Jong Young Choi, Mi-La Cho

**Affiliations:** ^1^The Rheumatism Research Center, Catholic Research Institute of Medical Sciences, The Catholic University of Korea, Seoul, Republic of Korea; ^2^Division of Hepatology, Department of Internal Medicine, College of Medicine, Seoul St. Mary's Hospital, The Catholic University of Korea, 505 Banpo-Dong, Seocho-Ku, Seoul 137-040, Republic of Korea; ^3^Division of Rheumatology, Department of Internal Medicine, The Catholic University of Korea, Seoul 137-040, Republic of Korea

## Abstract

T helper (Th) 17 cells are a subset of Th cells expressing interleukin- (IL-) 17 and initiating an inflammatory response in autoimmune diseases. Graft-versus-host disease (GVHD) is an immune inflammatory disease caused by interactions between the adaptive immunity of donor and recipient. The Th17 lineage exhibits proinflammatory activity and is believed to be a central player in GVHD. IL-1 performs a key function in immune responses and induces development of Th17 cells. Here, we show that blockade of IL-1 signaling suppresses Th17 cell differentiation and alleviates GVHD severity. We hypothesized that the IL-1 receptor antagonist (IL-1Ra) would suppress Th17 cell differentiation* in vitro* via inhibition of glycolysis-related genes. Blockade of IL-1 using IL-1Ra downregulated Th17 cell differentiation, an alloreactive T cell response, and expression of genes of the glycolysis pathway. Severity of GVHD was reduced in mice with a transplant of IL-Ra-treated cells, in comparison with control mice. To clarify the mechanisms via which IL-1Ra exerts the therapeutic effect, we demonstrated* in vivo* that IL-1Ra decreased the proportion of Th17 cells, increased the proportion of FoxP3-expressing T regulatory (T_reg_) cells, and inhibited expression of glycolysis-related genes and suppressed Th17 cell development and B-cell activation. These results suggest that blockade of IL-1 signaling ameliorates GVHD via suppression of excessive T cell-related inflammation.

## 1. Introduction

Interleukin- (IL-) 1 is a proinflammatory cytokine that drives an inflammatory response through IL-1 receptor signaling. For example, IL-1 is known to play an important role in the pathogenesis of metabolic inflammatory disorders [1]. Moreover, IL-1 triggers a self-amplifying cytokine network. IL-1 induces expression of inflammatory cytokines, and IL-1 signaling enhances differentiation into Th17 cells [2, 3]. Thus, IL-1 receptor antagonist (IL-1Ra) may be useful as an anti-inflammatory agent in inflammatory T cell-mediated diseases. Additionally, IL-1 is involved in the glycolysis pathway; various studies have shown that IL-1 is an important factor for upregulation of glucose uptake and glycolysis [4, 5].

Graft-versus-host disease (GVHD), the leading cause of morbidity and mortality associated with an allogeneic hematopoietic cell transplant, is a complex illness involving dysregulation of inflammatory cytokine cascades and distortion of the donor's cellular response to host alloantigens. Activation of alloreactive donor T cells is initiated by host antigen-presenting cells (APCs) including dendritic cells. Thus, T cells have been suggested as immunocompetent cells that cause GVHD [6], especially because Th17 cells contribute to the development of GVHD [7]. In addition, APCs play a significant role in the pathogenesis of GVHD; evidence shows that inactivation of APCs alleviates GVHD [8–10].

Th17 cells produce IL-17 and can lead to an autoimmune disease by activating an inflammatory response and innate immunity. There is a general consensus that Th17 cells control inflammation status and autoimmune diseases [11, 12]. Th17 cells are also involved in glucose and amino acid metabolism; the latter processes require Th17 cells [13], and hypoxia-induced factor-1*α*-dependent glycolysis activates differentiation into Th17 cells [14].

Blockade of IL-1 signaling is an effective therapeutic strategy against inflammatory disorders; it has been suggested that the inhibition of IL-1 signaling suppresses inflammation, and IL-1 antagonists are used as therapeutic agents in autoimmune diseases [15–17]. Nevertheless, there is a controversy regarding the therapeutic effects of IL-1 antagonists in GVHD [18, 19]. The aim of the present study was to determine the efficacy and mechanism of action of IL-1Ra treatment in acute GVHD. In this study, we performed* in vivo* and* in vitro* experiments to identify the effects and mechanisms of IL-1Ra activity during the development of acute GVHD in a mouse model.

## 2. Methods

### 2.1. Animals

Eight- to 10-week-old C57BL/6 (H-2k^b^, termed B6) and BALB/c (H-2k^d^) mice were purchased from Orient Bio (Sungnam, Korea). Foxp3-GFP knock-in mice (C57BL/6 strain) were purchased from Jackson Laboratories. The mice were maintained under specific pathogen-free (SPF) conditions at an animal facility with controlled humidity (55 ± 5%), light (12/12 h light/dark), and temperature (22 ± 1°C). The air at the facility was passed through a high-efficiency particulate arrestance (HEPA) filter system designed to exclude bacteria and viruses. The animals were fed standard mouse chow and tap water* ad libitum*. The protocols used in this study were approved by the Animal Care and Use Committee of the Catholic University of Korea.

### 2.2. The Bone Marrow Transplant (BMT) Model and Histopathological Analysis

After lethal irradiation (800 cGy), recipient (BALB/c) mice were injected intravenously (i.v.) with total bone marrow cells from donor mice. To induce acute GVHD, we isolated splenocytes from the donor mice, and then the splenocytes (1 × 10^7^) from MHC major and minor antigen-disparate B6 donors were incubated with IL-1Ra (anakinra, 50 ng/mL) or with vehicle (control) for 2 h at 37°C before adoptive transfer into the recipient mice. The clinical severity of GVHD was assessed twice a week using a scoring system consisting of five clinical parameters: weight loss, posture, activity, fur texture, and skin integrity [20]. The mice were euthanized on day 14 after the BMT for blinded histopathological analysis of GVHD-affected organs (skin and small intestine). The organs were harvested, cryoembedded, and sectioned on a cryotome. The tissue slices were fixed in 10% buffered formalin and stained with hematoxylin and eosin (H&E) for histological examination.

### 2.3. Cell Culture and Experimental Treatment

CD4^+^T cells were isolated from the spleen using CD4^+^T cell isolation kits according to the manufacturer's instructions. The purity of the isolated CD4^+^T cells was >95%. These cells were stimulated with a plate-immobilized anti-CD3 antibody (0.5 *μ*g/mL) and a soluble anti-CD28 antibody (1 *μ*g/mL) for 72 h in 24-well plates. Th17 cell development was induced by treatment with anti-interferon- (IFN-) *γ* (4 *μ*g/mL) and anti-IL-4 (4 *μ*g/mL) antibodies, TGF-*β* (2 ng/mL), and IL-6 (20 ng/mL) for 72 h. Aliquots of 10^5^ CD4^+^T cells (responders) were cultured with 10^5^ irradiated (2500 cGy) APCs in 96-well plates containing 200 *μ*L of the complete medium at 37°C in a humidified atmosphere containing 5% CO_2_ and were then pulsed with 1 *μ*Ci of [^3^H]TdR for 18 h before harvesting and counted using an automated harvester.

### 2.4. Flow Cytometry

To analyze intracellular cytokines, we stained splenocytes with PerCP-conjugated anti-CD4, APC-conjugated anti-CD25, FITC-conjugated anti-IL-17, and PE-conjugated anti-Foxp3 antibodies (eBiosciences), followed by fixation and permeabilization using a Foxp3 Staining Buffer Kit (BD Bioscience). Four hours before the staining, the cells were stimulated with phorbol myristate acetate (25 ng/mL) and ionomycin (250 ng/mL) (all from Sigma-Aldrich) and then treated with GolgiStop (BD Bioscience). All data were analyzed in the FlowJo software (Tree Star, Ashland, OR, USA).

### 2.5. Real-Time Quantitative PCR

The mRNA expression levels were estimated using a LightCycler 2.0 instrument (Roche Diagnostic, Mannheim, Germany) with version 4.0 software. All reactions were performed using the LightCyclerFastStart DNA Master SYBR Green I Kit (Takara, Shiga, Japan). The mRNA expression was normalized to that of *β*-actin. The primer sequences are shown in Table 1.

### 2.6. The Enzyme-Linked Immunosorbent Assay (ELISA)

The concentrations of IL-17, IFN-*γ*, TGF-*β*, and IL-10 were measured using sandwich ELISA (R&D Systems). Serum levels of IgG and IgG_1_ antibodies were measured using a commercially available ELISA kit (Bethyl Laboratories, Montgomery, TX, USA).

### 2.7. Statistical Analysis

All data were expressed as mean ± standard deviation (SD). The experimental data are presented as mean ± SD of triplicate cell culture experiments and are representative of the three independent experiments (not simultaneous). Statistical significance was assessed using the Mann-Whitney *U* test or analysis of variance (ANOVA) with Bonferroni's* post hoc* test using the GraphPad Prism software (v.5.01). *P* < 0.05 was assumed to denote statistical significance.

## 3. Results

### 3.1. Regulation of Th17 Cell Development and Expression of Genes Related to Glycolysis

**Figure 1:**
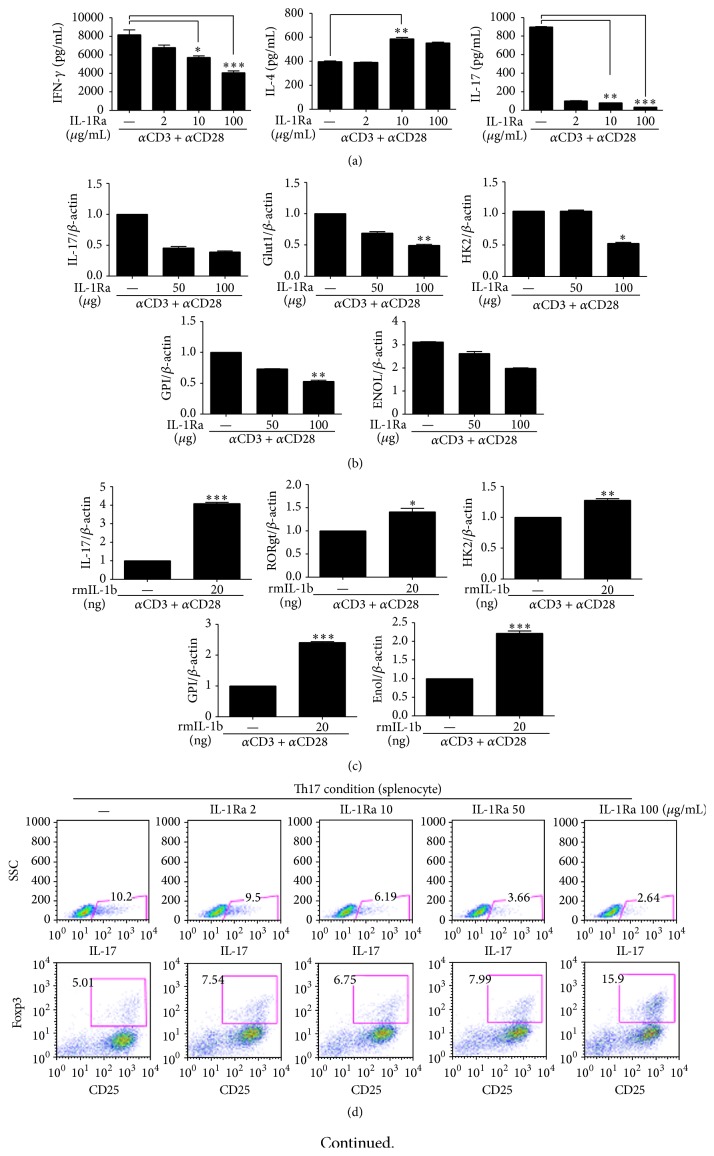
IL-1*β* controls the glycolysis pathway and differentiation into T helper (Th) 17 cells. Splenocytes from C57BL/6 mice were activated in the Th0 condition in the presence or absence of either IL-1 receptor antagonist (IL-1Ra) or recombinant IL-1*β* for 3 days. (a) The concentrations of interferon- (IFN-) *γ*, IL-4, and IL-17 in culture supernatants were measured by means of enzyme-linked immunosorbent assays (ELISAs). ((b) and (c)) The mRNA levels of IL-17, ROR*γ*t, and glycolysis-related factors were quantified using real-time PCR. Splenocytes from C57BL/6 mice were cultured under Th17-polarizing conditions for 3 days in the presence or absence of IL-1Ra. (d) The proportion of CD4^+^IL-17^+^ cells or CD4^+^CD25^+^Foxp3^+^ cells was determined using intracellular flow cytometric analysis. (e) The mRNA expression of IL-17, IL-21, Runx1, ROR*γ*t, Glut1, HK2, GPI, and MNOL was quantified using real-time PCR. ^*∗*^ *P* < 0.05, ^*∗∗*^ *P* < 0.01, and ^*∗∗∗*^ *P* < 0.001. Data are representative of 2 independent experiments.

Total splenocytes from normal C57BL/6 mice were cultured with anti-CD28 and anti-CD3 antibodies in the presence or absence of IL-1Ra. This molecule inhibited differentiation into Th17 cells in a dose-dependent manner; IL-17 concentration in the culture supernatant was significantly decreased by the IL-1Ra treatment (Figure 1(a)). IL-1Ra also inhibited secretion of IFN-*γ* into the culture supernatant in the Th0 condition. On the other hand, IL-4 secretion into the culture medium was enhanced significantly (Figure 1(a)). IL-1Ra inhibited expression of IL-17- and glycolysis-associated genes (Figure 1(b)). On the other hand, IL-1*β* treatment induced mRNA expression of IL-17, ROR*γ*t, and genes involved in glycolysis such as* HK2* (Figure 1(c)). To determine whether IL-1Ra inhibits differentiation into Th17 cells* in vitro*, we cultured the spleen cells under Th17-polarizing conditions in the presence of IL-1Ra. Differentiation into IL-17-expressing CD4^+^T cells, mainly Th17 cells, was suppressed significantly, whereas differentiation into CD4^+^CD25^+^Foxp3^+^T_reg_ cells was enhanced in a dose-dependent manner by IL-1Ra (Figure 1(d)). In addition, IL-1Ra inhibited expression of the Th17- and glycolysis-associated genes and of IL-17, IL-21, and ROR*γ*t genes (Figure 1(e)).

### 3.2. Inhibition of Th17 Cell Development Reduced Expression of Genes Involved in Glycolysis

**Figure 2:**
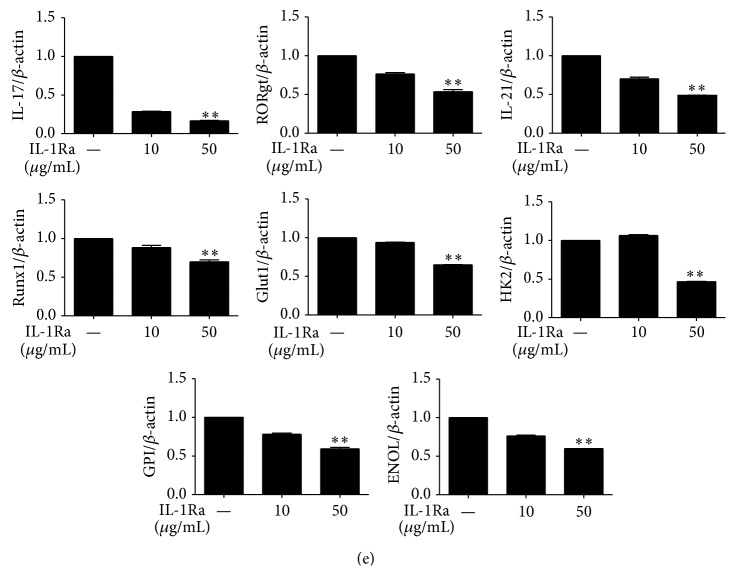
Treatment with IL-1Ra inhibits Th17 cell development via downregulation of the glycolysis pathway. Splenic CD4^+^T cells from C57BL6 mice were cultured under Th17-polarizing conditions in the presence or absence of IL-1Ra for 3 days. (a) The proportion of Th17 cells or T_reg_ cells was determined using flow cytometric analysis. (b) The supernatants were collected, and an enzyme-linked immunosorbent assay (ELISA) was performed to quantify the production of TGF-*β* and IL-17. ((c) and (d)) The mRNA levels of IL-17 and glycolysis-related factors and enzymes were measured using real-time PCR. ^*∗*^ *P* < 0.05, ^*∗∗*^ *P* < 0.01, and ^*∗∗∗*^ *P* < 0.001. Data are representative of 2 independent experiments.

To determine whether blockade of IL-1 signaling in T cells can inhibit differentiation into Th17 cells, we cultured CD4^+^T cells (that were isolated from the spleen) under Th17-polarizing conditions in the presence of IL-1Ra. IL-1Ra dose-dependently inhibited Th17 cell development and promoted differentiation into T_reg_ cells in the culture of mouse splenocytes under Th17 induction conditions (Figure 2(a)). In addition, IL-1Ra inhibited the production of IL-17 but enhanced TGF-*β* production (Figure 2(b)). The mRNA levels of IL-17 and glycolysis-associated genes were reduced dose-dependently by the IL-1Ra treatment (Figure 2(c)). Gene expression of glycolysis factors including triosephosphate isomerase (TPI) and lactate dehydrogenase A (LDL*α*) was also decreased significantly by IL-1Ra treatment (Figure 2(d)). These results suggested that the blockade of IL-1 receptor drove the development of Th17 cells and T_reg_ cells in opposite directions.

### 3.3. Attenuation of the Alloreactive T Cell Response

**Figure 3:**
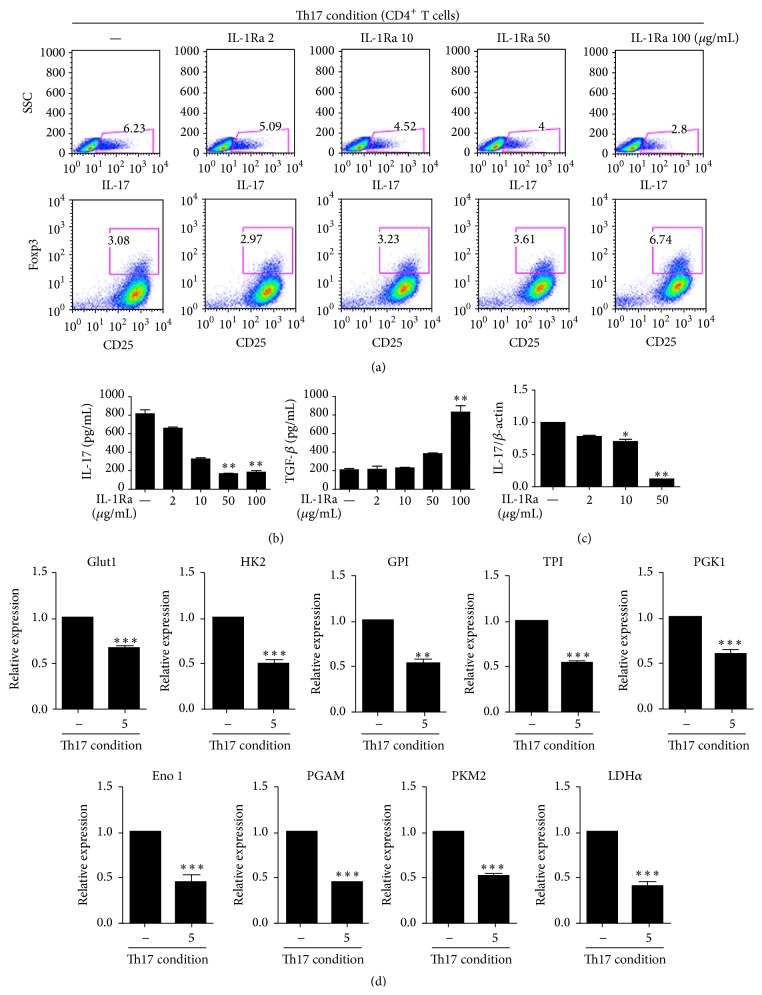
Treatment with IL-1 receptor antagonist (IL-1Ra) reduces the alloreactive T cell response. Antigen-presenting cells (APCs) from C57BL/6 mice (an allogeneic stimulator) were cocultured with T cells from BALB/c mice (responder cells) and subjected to the indicated stimuli for 3 days. (a) The proliferation of alloreactive T cells was quantified by MLR. (b) The concentrations of IL-17 and interferon- (IFN-) *γ* in the culture supernatants were measured using enzyme-linked immunosorbent assays (ELISAs). (c) Flow cytometric assessment of the numbers of Th1 cells and Th17 cells. ^*∗*^ *P* < 0.05, ^*∗∗*^ *P* < 0.01, and ^*∗∗∗*^ *P* < 0.001. Data are representative of 2 independent experiments.

To determine the impact of IL-1 receptor blockade on the proliferative capacity of donor CD4^+^T cells in response to alloantigens, we measured T cell alloreactivity after treatment with IL-1Ra by means of [^3^H]thymidine incorporation. After 3 days, CD4^+^T cells proliferated excessively in response to allogeneic APCs. In contrast, treatment with IL-1Ra resulted in a potent dose-dependent inhibition of the proliferation of the alloreactive T cells (Figure 3(a)). The elevation of IFN-*γ* and IL-17 concentrations in the culture supernatant was also attenuated by the IL-1Ra treatment in a dose-dependent manner (Figure 3(b)). In addition, the IL-1Ra treatment reduced the population of Th1 cells and Th17 cells in a dose-dependent manner (Figure 3(c)). These data showed that IL-1Ra was an effective regulator of the alloreactive-CD4^+^T cell response.

### 3.4. Alleviation of GVHD by a Transplant of Donor Cells with Blocked IL-1 Signaling

**Figure 4:**
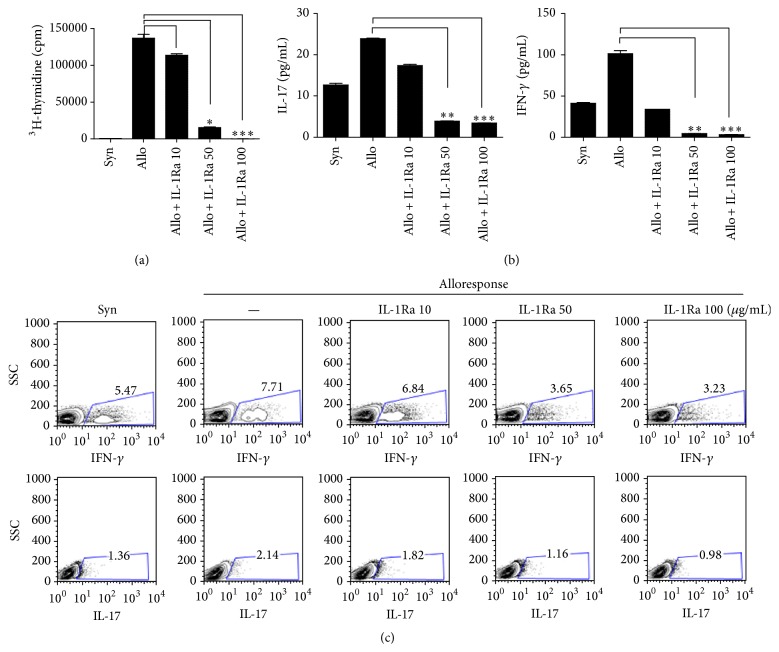
Blockade of IL-1 signaling in donor cells inhibits severity of acute graft-versus-host disease (GVHD). (a) Splenocytes (from C57BL/6 mice) that were cultured with or without IL-1 receptor antagonist (IL-1Ra) for 2 h were transplanted into recipient mice (BALB/c), and the clinical features of acute GVHD were then monitored (*n* = 5 per group). (b) Twelve days after the bone marrow transplant (BMT), histopathological analysis was performed on the skin and small intestine. ^*∗*^ *P* < 0.05. Data are representative of 2 independent experiments.

To test whether blockade of IL-1 signaling has therapeutic effects on GVHD, a BMT was performed using splenocyte culture in the presence or absence of IL-1Ra. Animals with acute GVHD who received a transplant of IL-1Ra-treated splenocytes showed a reduction of severity of acute GVHD and attenuation of weight loss, in comparison with the animals in the control GVHD group (Figure 4(a)). In addition, we compared the histopathological features of GVHD-affected organs. In recipient mice that received a transplant of IL-1Ra-treated donor cells, the clinical severity of acute GVHD affecting the skin and small intestine was reduced. These findings were suggestive of decreased lymphocyte infiltration, inflammation, and fibrosis in comparison with control mice (Figure 4(b)).

### 3.5. Analysis of B Cells and CD4^+^T Cells in IL-1Ra-Treated Mice with GVHD

**Figure 5:**
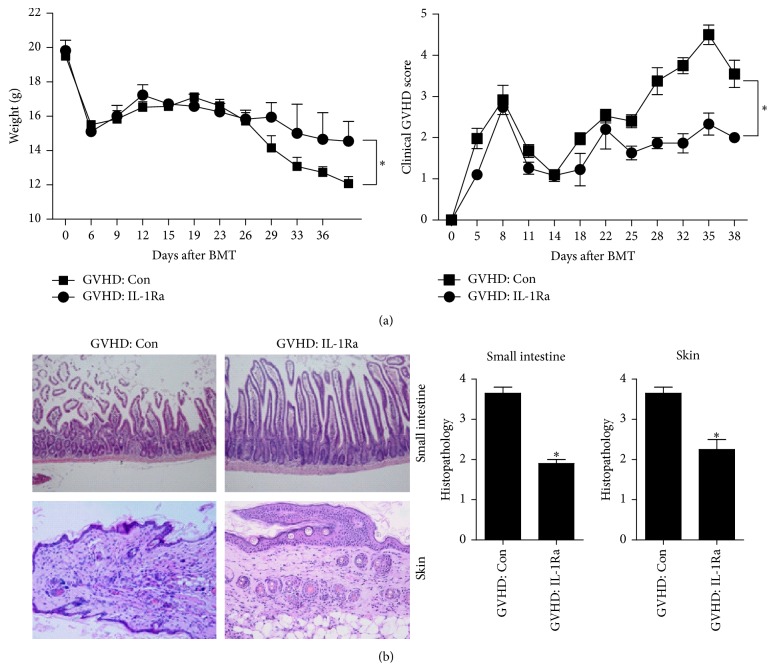
Analysis of B cells and CD4^+^T cells in mice with acute graft-versus-host disease (GVHD), in whom splenocytes were treated with IL-1 receptor antagonist (IL-1Ra): (a) to elucidate the* in vivo* mechanism of action of the blockade of IL-1 signaling in amurine model of acute GVHD, we analyzed the proportion of Th1, Th2, and Th17 and (b) shows T_reg_ cells among* ex vivo* splenocytes from each group by means of flow cytometry. (c) The share of the B-cell subset was analyzed using flow cytometry. B220^+^ B cells included IgM^high^IgD^low^ (immature B cells) and IgM^low^IgD^low^ (mature B cells). (d) shows The level of IgG in serum. ^*∗*^ *P* < 0.05, ^*∗∗*^ *P* < 0.01. Data are representative of 2 independent experiments.

To elucidate the* in vivo* mechanism of action of IL-1Ra in the murine model of acute GVHD, we used fluorescence-activated cell sorting (FACS) to count the Th1, Th2, Th17, and T_reg_ cells in spleens isolated from each group of mice. The percentages of IFN-*γ*-producing CD4^+^T cells and IL-17-producing CD4^+^T cells in spleens were lower in IL-1Ra-treated GVHD animals than in the GVHD control group (Figure 5(a)). In contrast, the numbers of IL-4-producing CD4^+^T cells and CD4^+^CD8^+^Foxp3^+^T_reg_ cells were significantly higher in the IL-1Ra-treated group. To determine whether there was a change in B cell subpopulations after the IL-1Ra treatment, we analyzed splenocytes from the mice with acute GVHD. Differentiation into mature B cells in GVHD was suppressed significantly in the IL-1Ra group compared to controls. On the other hand, differentiation into immature B cells was increased significantly in the GVHD IL-1Ra group compared to controls (Figure 5(c)). Concentrations of IgG and IgG_1_ decreased in the serum of IL-1Ra-treated animals compared to the GVHD control group (Figure 5(d)).

## 4. Discussion

Blockade of IL-1 signaling can reduce inflammation, which makes IL-1Ra an important target of research in inflammatory diseases. Nonetheless, the effects of IL-1Ra in GVHD are not clear [18, 19]. However, IL-1Ra is involved in GVHD pathogenesis. Indeed, IL-1Ra expression in saliva of GVHD patients was decreased significantly compared to normal controls [21]. Recently, IL-1 receptor deficiency in dendritic cells and T cells ameliorates acute GVHD enhancing survival [22]. It is also documented that IL-1 blockade could be effective in reducing GVHD development [23]. Our study shows that IL-1Ra inhibits Th17 cell development and the alloreactive T cell response through inhibition of the glycolysis pathway. Additionally, we confirmed alleviation of severity and the immune response in GVHD by a transplant with IL-1Ra-treated splenocytes. This therapeutic effect and the apparent mechanism of the IL-1Ra treatment are the most substantial findings of our study.

Each subset of T cells plays a specific role in adaptive immunity. It is well known that Th1 cells and Th17 cells activate immunity and inflammation, whereas T_reg_ cells inhibit the development of Th1 cells and Th17 cells, thereby limiting redundant inflammatory responses [24]. Moreover, the Th17/T_reg_ ratio plays an important role in GVHD. There is evidence that the Th17/T_reg_ ratio in the peripheral blood of patients with GVHD is significantly higher in comparison with healthy controls, suggesting that the Th17/T_reg_ ratio can be used as a sensitive and specific biomarker of GVHD [25]. According to our data, IL-1Ra treatment reduces differentiation into Th1 and Th17 cells while inducing differentiation into T_reg_ cells. These results indicate that IL-1Ra may have a therapeutic value in GVHD.

The Th17 lineage has been recognized as the activator of proinflammatory responses; in particular, Th17 cells perform a pivotal function in inflammation in many autoimmune diseases including GVHD [26, 27]. IL-4 that is produced by Th2 cells exerts a coordinated anti-inflammatory activity by inhibiting IL-1 expression and by upregulating IL-1Ra [28, 29]. In the present study, Th17 cell development is stimulated by IL-1*β* treatment. On the other hand, IL-1Ra inhibits differentiation into Th1 cells and Th17 cells while inducing Th2 cell development in our mouse model of GVHD. Thus, IL-Ra may be used to inhibit T cell-related inflammation and to enhance an anti-inflammatory response.

GVHD is characterized by weight loss and selective damage to several organs including the skin and gastrointestinal tract. In GVHD patients, these organs are damaged predominantly [30]. It is also known that weight loss usually occurs in patients with GVHD [31]. Moreover, a skin biopsy is necessary for diagnosis of GVHD after an intestinal transplant [32]. We demonstrate here that IL-Ra treatment suppresses weight loss and decreases tissue damage in a mouse model of GVHD. Therefore, IL-1Ra may be a promising therapeutic agent for GVHD.

Alloreactive T cells take part in the pathogenesis of GVHD. It has been suggested that expansion and development of alloreactive T cells contribute to the development of GVHD [33]. Inhibition of differentiation into alloreactive T cells suppresses preexisting GVHD; for example, an inhibitor of proliferation of alloreactive T cells (inducing apoptosis) slows down the development of GVHD [34]. Our study shows that IL-1Ra inhibits the alloreactive T cell response and the production of IFN-*γ* and IL-17* in vitro*. These results indicate that IL-1Ra may stop the progression of GVHD.

Glycolysis is known to be involved in Th17 cell development. Because suitable energy precursors and synthetic precursors are necessary for activation of T cells, during this process, glucose uptake and glycolysis are enhanced, as are amino acid transport and glutaminolysis [35–40]. Specific metabolic pathways are needed to activate different T cell subsets in order to utilize their unique activities in immunity and an inflammatory response. For example, lipid oxidation enhances T_reg_ cell development and reduces the activity and endurance of Th17 cells [41]. In addition, glucose uptake and expression of genes that are involved in glycolysis (such as* Glut1*) are induced in Th17 cells compared to T_reg_ cells [41]. It is also known that aerobic glycolysis in response to hypoxia induces differentiation into Th17 cells, thus regulating the Th17/T_reg_ balance [14].

Although IL-1 blockade revealed nontherapeutic effect in GVHD patients [18], IL-1 exacerbated the severity of GVHD in murine model [23]. Additionally, GVHD related mortality was decreased by receptor antagonism or depletion of IL-1*β* [22]. Thus, clinical trial in GVHD patients will be needed to confirm the therapeutic effect of IL-1 blockade.

In the present study, IL-1*β* stimulates Th17 cell development by upregulating the glycolysis pathway. On the other hand, IL-1Ra suppresses differentiation into Th17 cells, while upregulating T_reg_ cells through inhibition of the glycolysis pathway. The therapeutic properties of IL-1Ra can be explained by downregulation of Th17 cells via inhibition of glycolysis.

The observations pointing to the anti-inflammatory effects of IL-1Ra open up new possibilities with respect to treatment of GVHD. We believe that IL-1Ra induces T_reg_ cell development and downregulates the Th17 cells, thereby reducing an inflammatory response through inhibition of the glycolysis pathway in Th17 cells. This observational evidence demonstrates that IL-1Ra is a strong candidate for a new therapeutic agent against GVHD.

## Figures and Tables

**Table 1:** PCR primers used in this study.

Gene	Sense primer (5′→3′)	Antisense primer (3′→5′)	PCR product size (bp)
IL-4	CGA GTA ATC CAT TTG CAT GAT GC	ACG GAG ATG GAT GTG CCA AAC GTC	279
IL-17	CCT CAA AGC TCA GCG TGT CC	GAG CTC ACT TTT GCG CCA AG	101
Glut1	CAGTTCGGCTATAACACTGGTG	GCCCCCGACAGAGAAGATG	156
HK2	TGATCGCCTGCTTATTCACGG	AACCGCCTAGAAATCTCCAGA	112
GPI	TCAAGCTGCGCGAACTTTTTG	GGTTCTTGGAGTAGTCCACCAG	105
Eno1	TGCGTCCACTGGCATCTAC	CAGAGCAGGCGCAATAGTTTTA	118
RORgt	TGT CCT GGG CTA CCC TAC TG	GTG CAG GAG TAG GCC ACA TT	188
IL-21	CCC TTG TCT GTC TGG TAG TCA TC	ATC ACA GGA AGG GCA TTT AGC	347
Rnux1	TAC CTG GGA TCC ATC ACC TC	GAC GGC AGA GTA GGG AAC TG	164
TPI	CCAGGAAGTTCTTCGTTGGGG	CAAAGTCGATGTAAGCGGTGG	144
PGK1	ATGTCG CTTTCCAACAAGCTG	GCTCCATTGTCCAA GCAGAAT	164
PGAM	TCTGTGCAGAAGAGAGCAATCC	CTGTCA GACCGCCATAGTGT	118
PKM2	GCCGCCTGGACATTGACTC	CCATGAGAGAAATTCAGCCGAG	145
LDH*α*	CATTGTCAAGTACAGTCCACACT	TTCCAATTACTCGGTTTTTGGGA	113
